# Non-Alcoholic Fatty Liver Disease as an Emerging Risk Factor for Heart Failure

**DOI:** 10.1007/s11897-023-00613-1

**Published:** 2023-07-04

**Authors:** Riccardo M. Inciardi, Alessandro Mantovani, Giovanni Targher

**Affiliations:** 1 ASST Spedali Civili Di Brescia, Division of Cardiology and Department of Medical and Surgical Specialties, Radiological Sciences and Public Health, University of Brescia, Brescia, Italy; 2 Department of Medicine, Section of Endocrinology, Diabetes, and Metabolism, University of Verona, Verona, Italy

**Keywords:** NAFLD, Non-alcoholic fatty liver disease, Heart failure, Metabolic dysfunction-associated fatty liver disease, Cardiovascular disease

## Abstract

**Purpose of the Review:**

Non-alcoholic fatty liver disease (NAFLD) and heart failure (HF) are two chronic diseases that have become important global public health problems. This narrative review provides a comprehensive overview of the association between NAFLD and increased risk of new-onset HF, briefly discusses the putative biological mechanisms linking these two conditions, and summarizes targeted pharmacotherapies for NAFLD that might also beneficially affect cardiac complications leading to new-onset HF.

**Recent Findings:**

Recent observational cohort studies supported a significant association between NAFLD and the long-term risk of new-onset HF. Notably, this risk remained statistically significant even after adjustment for age, sex, ethnicity, adiposity measures, pre-existing type 2 diabetes and other common cardiometabolic risk factors. In addition, the risk of incident HF was further increased with more advanced liver disease, especially with higher severity of liver fibrosis. There are multiple potential pathophysiological mechanisms by which NAFLD (especially in its more advanced forms) may increase the risk of new-onset HF.

**Summary:**

Because of the strong link existing between NAFLD and HF, more careful surveillance of these patients will be needed. However, further prospective and mechanistic studies are required to better decipher the existing but complex link between NAFLD and risk of new-onset HF.

## Introduction

Congestive heart failure (HF) and non-alcoholic fatty liver disease (NAFLD) are two growing major clinical and public health problems globally [1, 2].

Convincing evidence indicates that the clinical burden of NAFLD is not only restricted to its liver-related complications [such as non-alcoholic steatohepatitis (NASH), advanced fibrosis, cirrhosis or hepatocellular carcinoma] but also adversely affects multiple extrahepatic organs and systems, including the heart and vascular system [3–5]. In particular, NAFLD is not only associated with a substantially higher risk of developing major adverse cardiovascular events (that are the leading cause of mortality in people with NAFLD) [6, 7], but is also associated with a higher risk of cardiac arrhythmias (mainly atrial fibrillation) and myocardial remodeling, which may precede and/or promote the development of new-onset HF [8, 9•, 10]. So, because of the close link between NAFLD and HF, more careful surveillance of these patients is needed.

This narrative review article focuses on the most recent observational cohort studies supporting a significant association between NAFLD and the risk of developing new-onset HF. We also discuss the epidemiology and diagnosis of NAFLD, the putative biological mechanisms underpinning the association between NAFLD and risk of new-onset HF, and briefly summarize targeted pharmacotherapies for NAFLD or NASH that may also beneficially affect cardiac complications leading to new-onset HF over time.

## Epidemiology and Diagnosis of NAFLD

### NAFLD Epidemiology

NAFLD has become the most common cause of chronic liver disease worldwide, affecting up to nearly 30% of adults in the general population [11]. The prevalence of non-alcoholic steatohepatitis (NASH) in the general adult population is challenging to estimate precisely [12]. Based on the currently available data, the global prevalence of NASH is estimated between ~ 2% and 6% in the general adult population [11]. The global prevalence rates of NAFLD and NASH markedly increase in specific patient populations, such as patients who are obese or have type 2 diabetes mellitus (T2DM) [3]. For instance, in a 2019 systematic review and meta-analysis including 80 observational studies for a total of approximately 49,500 individuals with T2DM, Younossi et al*.* reported that the estimated global prevalence of NAFLD (as detected by liver ultrasonography or magnetic resonance spectroscopy) amongst patients with T2DM was 55.5% (95% CI 47–64%); studies from Europe reported the highest prevalence (68% [95% CI 62–73%]) [13]. In addition, the authors also found that the global prevalence rates for NASH and advanced fibrosis (stage ≥ F3 on liver histology) among T2DM patients were 37% and 17%, respectively [13]. In a 2023 meta-analysis including 151 observational studies for a total of 101,028 overweight and obese individuals, Quek et al*.* reported that in the overweight population the estimated global prevalence of NAFLD and NASH was approximately 70% and 33%, respectively [14]. Similarly, in the obese population the global prevalence of NAFLD and NASH was 75% and 34% [14]. Additionally, the estimated global prevalence of advanced fibrosis was around 7% among overweight and obese individuals [14]. Differences in terms of the prevalence of NAFLD may also exist in relation to sex and ethnicity. For example, the global prevalence of NAFLD is higher in men than in premenopausal women, but tends to be comparable between men and postmenopausal women of similar age [15]. Hispanic and Caucasian individuals are more likely to have NAFLD, while African Americans are at a lower risk for NAFLD [16].

Although detailed information about the incidence rates of NAFLD is currently lacking, a recent systematic review and meta-analysis of 63 observational studies (~ 1,200,000 participants) showed that the global incidence of NAFLD was about 46 cases per 1000 person-years, with higher incidence rates observed in males and overweight/obese individuals compared to females and those of normal body weight [17].

### NAFLD Diagnosis

The NAFLD acronym includes a spectrum of progressive steatotic liver conditions, ranging from non-alcoholic fatty liver (NAFL) to NASH, advanced fibrosis and cirrhosis [12, 18•]. NAFL is defined histologically by the presence of macrovesicular steatosis in ≥ 5% of hepatocytes without evidence of hepatocyte injury (ballooning) in persons with no or little alcohol consumption [12, 18•]. NASH is defined histologically by the presence of ≥ 5% steatotic hepatocytes with coexisting inflammation and hepatocyte ballooning, independent of liver fibrosis [12, 18•]. Advanced fibrosis refers to histologic stages F3-F4 on the Kleiner’s classification that is the presence of either “bridging fibrosis” (F3 stage) or cirrhosis (F4 stage) [12, 18•, 19]. Currently, the diagnosis of NAFLD is always a diagnosis of exclusion that is mainly based on the following criteria: (a) presence of hepatic steatosis (detected by serum biomarkers/scores, imaging techniques or liver histology); (b) no significant alcohol consumption (conventionally defined as < 20 g/day for women and < 30 g/day for men); and (c) no other secondary causes of hepatic steatosis (e.g., virus, hemochromatosis, autoimmune hepatitis, alpha-1 anti-trypsin deficiency, Wilson’s disease or use of potentially hepatotoxic drugs) [12, 18•].

In 2020, international experts have proposed to change the terminology and definition of this common metabolic liver disease, switching from NAFLD to metabolic dysfunction-associated fatty liver disease (MAFLD) to overcome the intrinsic limitations of the NAFLD definition and to further highlight the pathogenic role of metabolic dysfunction in the development and progression of this liver disease [20, 21]. Based on this newly-proposed definition, the diagnosis of MAFLD is based on the coexistence of hepatic steatosis (detected by serum biomarkers/scores, imaging techniques or liver biopsy) and at least one of the following three metabolic risk abnormalities: (a) overweight or obesity, (b) T2DM, or (c) metabolic dysregulation (defined by the presence of at least two metabolic risk factors, typically featuring the metabolic syndrome, amongst increased waist circumference, high plasma triglycerides, low HDL cholesterol level, hypertension, prediabetes, insulin resistance [assessed by homeostasis model assessment of insulin resistance (HOMA-IR score) ≥ 2.5] or systemic low-grade inflammation [evaluated by a plasma high-sensitive C reactive protein level > 2 mg/L]) [20, 21]. Emerging data suggested that adopting the MAFLD definition (instead of NAFLD definition) more individuals with liver damage may be identified [22]. However, since there is not a global support for the newly proposed MAFLD definition yet, we have decided to use in this narrative review the term NAFLD instead of that of MAFLD.

Liver biopsy remains the gold standard method for diagnosing and staging NAFLD, as it is the only diagnostic method, which is able to differentiate between NAFL and NASH and to quantify liver fibrosis [12, 18•, 23, 24]. However, liver biopsy is invasive, patient-unfriendly, and potentially risky [12, 18•, 23, 24]. For these reasons, liver biopsy assessment is not used routinely for the diagnosis of NAFLD, but it is used sparingly in clinical practice [12, 18•, 23, 24]. Conventional liver ultrasonography is the recommended first-line imaging technique for the diagnosis of NAFLD (hepatic steatosis) in clinical practice [12, 18•, 23, 24]. This imaging method is inexpensive, patient friendly, and largely spread in several clinical settings [12, 18•, 23, 24]. However, liver ultrasonography is operator-dependent [12, 18•, 23, 24] and lacks sufficient sensitivity for accurately quantifying or monitoring changes in hepatic fat content [25]. Controlled attenuation parameter (CAP), in combination with vibration-controlled transient elastography (Fibroscan®), is another non-invasive method that can be used for the diagnosis of hepatic steatosis [26]. However, at present, the specific CAP thresholds for detecting hepatic steatosis are not yet established [23]. Computed tomography offers a semi-quantitative imaging method for detecting hepatic steatosis, but it also lacks sufficient sensitivity for smaller amounts of liver fat and exposes the subject to high radiation levels [12, 18•, 23]. Magnetic resonance imaging–proton density fat fraction (MRI-PDFF) and proton magnetic resonance spectroscopy have emerged as the two most accurate and reproducible imaging methods for the non-invasive quantification of liver fat content [24, 27]. However, both imaging methods are expensive and used only in clinical research or tertiary care centers [12, 18•, 23, 24]. Vibration-controlled transient elastography (Fibroscan®) is the most widely used method for non-invasively staging hepatic fibrosis in clinical practice, since it is broadly validated, patient-friendly and also provides real-time results [12, 18•, 23, 24]. Fibroscan® has a good reproducibility and excellent performance in identifying advanced fibrosis or cirrhosis [12, 18•, 23, 24]. However, its diagnostic performance is reduced by presence of severe obesity [12, 18•, 23, 24].

## NAFLD as Risk Factor for New-Onset HF

More than 10 years ago, the Framingham Heart Study and some other large community-based cohort studies from UK and Finland reported that higher serum gamma-glutamyltransferase concentrations within the "normal" range (as a surrogate marker of NAFLD) were associated with a higher risk of new-onset HF events, independently of daily alcohol consumption and a wide range of common risk factors for HF [28–30].

In 2021, using a nationwide health screening database of about 9 million middle-aged Korean individuals followed for a median of 10.1 years, Lee et al. reported that NAFLD (defined as fatty liver index [FLI] ≥ 30) was significantly associated with a higher risk of incident HF events (adjusted hazard ratio [HR] 1.61, 95% confidence interval [CI] 1.55–1.67). This association was independent of age, sex, household income, residential area, the Charlson’s comorbidity index, smoking history, physical activity, and estimated glomerular filtration rate [31]. Most interestingly, in a nationwide cohort study of 10,422 Swedish adult individuals with biopsy-confirmed NAFLD and nearly 50,000 matched control subjects who were followed for a median of 13.6 years, Simon et al. [32] examined the risk of incident major adverse cardiovascular events (including also the risk of new-onset HF), according to the presence and histological severity of NAFLD. These authors found that compared with matched population controls, patients with NAFLD had a significantly higher incidence of HF (adjusted HR 1.75, 95% CI 1.63–1.87) even after adjustment for common cardiometabolic risk factors. Rates of incident HF events increased progressively with worsening NAFLD severity, with the highest incidence rates observed with non-cirrhotic fibrosis (adjusted HR 2.04, 95% CI 1.66–2.51) and cirrhosis (adjusted HR 2.83, 95% CI 2.08–3.85) [32].

**Fig. 1:**
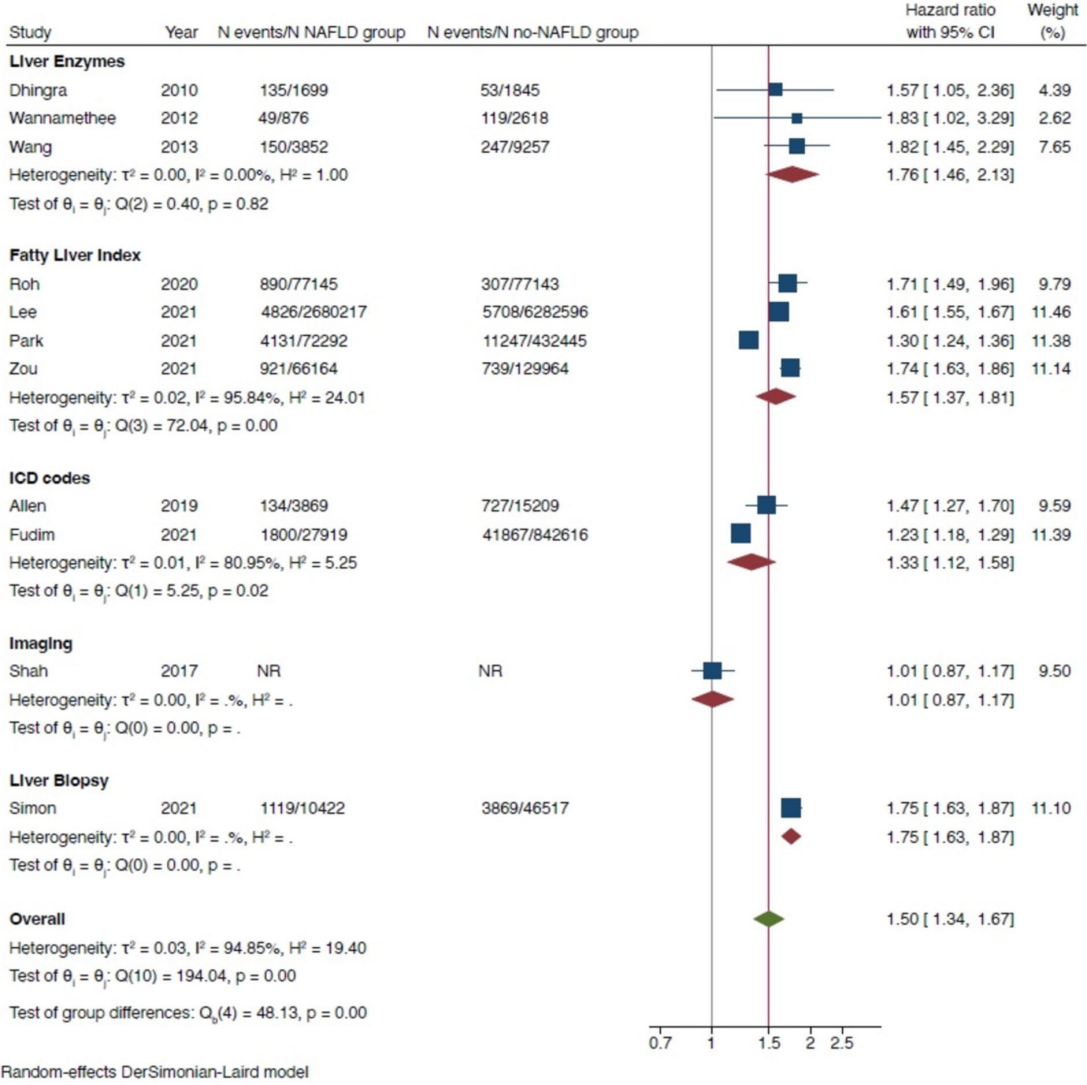
Forest plot and pooled estimates of the effect of NAFLD on the risk of new-onset heart failure in eleven eligible cohort studies, stratified by methodologies used for the diagnosis of NAFLD. Data are reproduced from Mantovani et al. [33•]

In 2023, we included the aforementioned longitudinal cohort studies in a comprehensive meta-analysis that incorporated a total of 11 observational cohort studies with more than 11 million middle-aged individuals from different countries and captured nearly 98,000 cases of new-onset HF over a median of 10-year follow-up [33•]. As shown in the forest plot of Fig. 1, our meta-analysis concluded that the presence of NAFLD (diagnosed by blood biomarkers/scores, International Classification of Diseases (ICD)-10 codes, imaging techniques, or liver histology) was significantly associated with a 1.5-fold higher risk of developing new-onset HF (pooled random-effects HR 1.50, 95% CI 1.34–1.67; p < 0.001). This risk remained significant even after adjustment for age, sex, ethnicity, adiposity measures, hypertension, T2DM and other cardiometabolic risk factors. In addition, the magnitude of this risk remained unchanged even when the comparison was stratified by study country, follow-up duration, modality of HF diagnosis or methods used for diagnosing NAFLD. Notably, the risk of incident HF appeared to increase further with greater severity of NAFLD, especially with higher fibrosis stage [33•]. These latter observations are also supported by recent longitudinal studies showing that increased fibrosis-4 (FIB-4) index or other non-invasive liver fibrosis scores were associated with a higher risk of hospitalization for HF (adjusted HR 2.09, 95% CI 1.86–2.35) in a large real-word cohort of patients with established NAFLD or NASH [34]. Similarly, these results are supported by data from cohorts of patients with chronic HF, especially in HF patients with preserved ejection fraction (HFpEF) [35, 36]. However, further studies are needed to prove whether the severity of liver disease in NAFLD further amplifies the risk of new-onset HF.

Interestingly, after the publication of our updated meta-analysis [33•], Simon et al. examined the association between NAFLD and risk of developing new-onset HF in a nationwide cohort study of 699 Swedish obese children and young adults ≤ 25 years old with histologically confirmed NAFLD and 3,353 control subjects matched for age, sex, calendar year and county [37]. Over a median follow-up of 16.6 years, these authors found that compared with matched population controls, young patients with NAFLD had significantly higher incidence rates of congestive HF (adjusted HR 3.89, 95% CI 1.20–12.6) that appeared to be further augmented with NASH. These findings suggest that research to better characterize cardiovascular risk also in obese children and young adults with NAFLD should be prioritized [37].

Based on the currently available data, there seems little doubt that NAFLD is associated with an increased incidence of HF, an association that has been consistently replicated across different countries, as well as across different methods used for NAFLD diagnosis. To date, little is known on the association between NAFLD and different HF phenotypes (i.e., HF with reduced left ventricular ejection fraction [HFrEF] vs. preserved left ventricular ejection fraction [HFpEF]) as most of the published cohort studies did not have any echocardiographic data to categorize LV ejection fraction. The only cohort study that examined this issue showed that the association of NAFLD with the risk of developing HF was stronger for HFpEF than for HFrEF [38]. This is also in line with previously published studies reporting a significant association of NAFLD with LV hypertrophy and subclinical LV diastolic dysfunction in the context of preserved ejection fraction [39, 40]. Notably, the association of NAFLD with impaired cardiac structure and function remained statistically significant even after adjusting for obesity and other common cardiometabolic risk factors, thus suggesting a possible direct pathophysiological link between NAFLD and the risk of HFpEF. Unlike HFrEF, HFpEF has distinct clinical phenotypes. Along with the obese-diabetic phenotype, which is the one often encountered in clinical practice, clinicians should be also aware of the potential coexistence of NAFLD or NASH in the context of HFpEF.

Collectively, therefore, the healthcare professionals should be aware that the risk of new-onset HF is moderately greater among patients with NAFLD, especially among those with fibrotic NASH. This further highlights the need for a multidisciplinary and holistic approach to manage both liver disease and cardiometabolic risk in patients with NAFLD [41]. However, as discussed in a later section, whether NAFLD is an independent risk factor for new-onset HF or it is simply a bystander that shares common cardiometabolic risk factors is still controversial.

## Putative Mechanisms Linking NAFLD to Risk of New-Onset HF

Although the exact pathophysiological mechanisms by which NAFLD may increase the risk of new-onset HF are not fully understood, it is possible to speculate that several factors play a key role. A detailed description of the putative mechanisms linking NAFLD to increased risk of HF has been extensively discussed elsewhere [9•]. Briefly, multiple factors related with coexisting obesity, T2DM or directly linked to intestinal dysbiosis might modulate the association between NAFLD and the risk of new-onset HF [8, 9•, 42]. For instance, systemic low-grade inflammation that typically characterizes metabolic disorders, such as obesity, T2DM and NAFLD [42], may contribute to the development of accelerated coronary atherosclerosis, as well as to the development of myocardial remodeling and hypertrophy, thereby promoting the onset of HF [8, 9•]. Specifically, proinflammatory cytokines, such as interleukin (IL)-1, IL-6, tumor necrosis factor-alpha or transforming growth factor-beta, may promote myocardial remodeling, myolysis and fibrosis via several mechanisms [43–46]. In this regard, accumulating evidence indicates a potential beneficial effect of IL-1 blockade in terms of cardiac contractility, quality-of-life, and treadmill exercise time, as well as in terms of reduction in serum NT-proBNP concentrations in patients with established HF [47]. Dietary fatty acids may also promote systemic low-grade inflammation and even alter gut microbiota composition [48–50]. On this subject, a high-fat Western diet induces endotoxaemia, which, in turn, promotes low-grade inflammation and production of specific microbial metabolites, including trimethylamine (TMA) or TMA N-oxide (TMAO) [48]. Interestingly, higher circulating levels of TMA and TMAO are associated with the future risk of adverse cardiovascular outcomes [51].

**Fig. 2:**
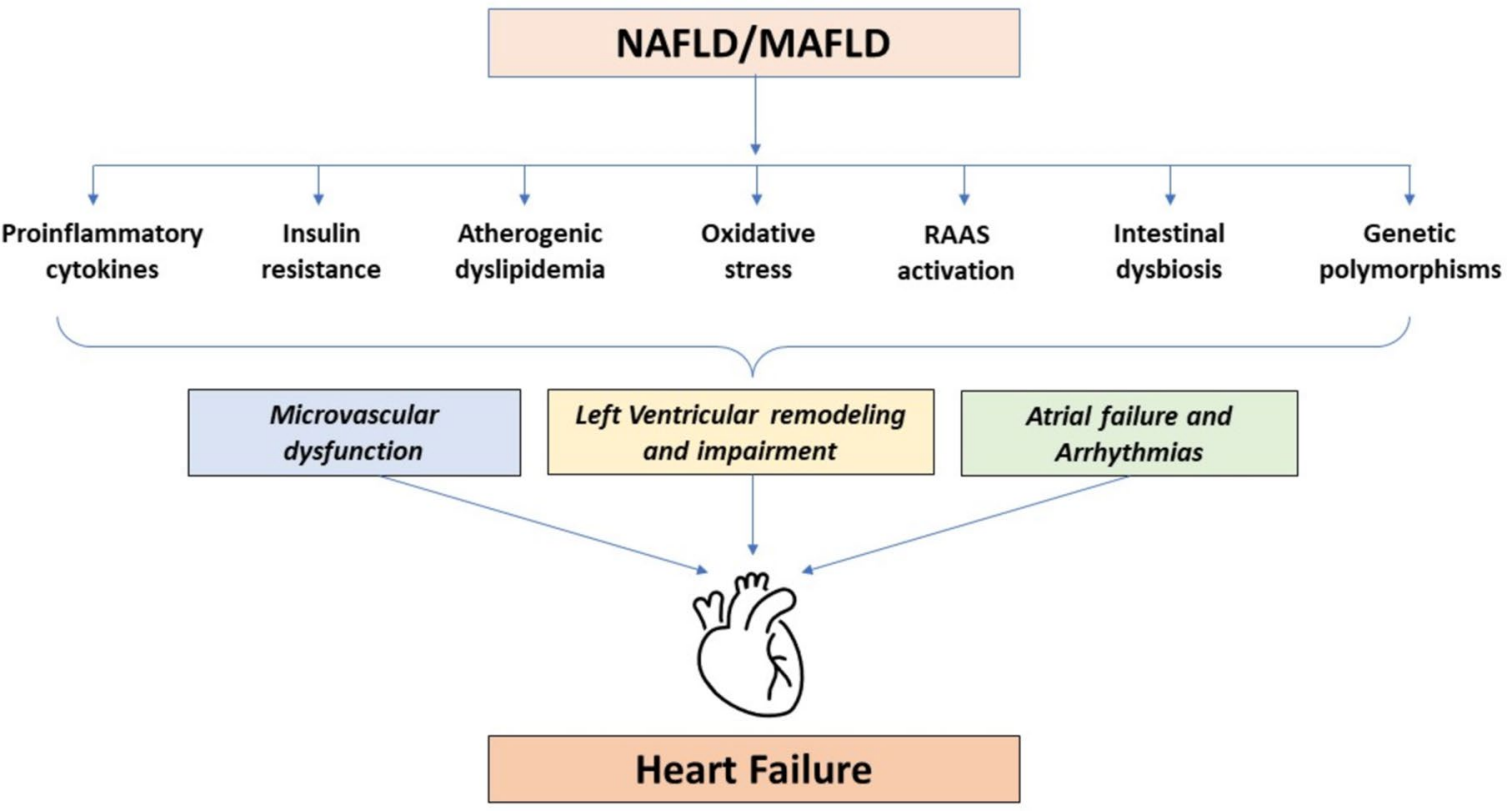
Putative pathophysiological mechanisms underlying the association between NAFLD and risk of new-onset heart failure. In NAFLD, many alterations occur within the liver, resulting in an increased production of proinflammatory cytokines, worsening of insulin resistance, promotion of a more pronounced atherogenic lipid profile, exacerbation of oxidative stress, activation of the renin–angiotensin-aldosterone system (RAAS) and alteration of gut microbiota, accompanied by an increased production of bioactive microbial metabolites. All these factors, along with specific genetic polymorphisms, may lead microvascular dysfunction, myocardial remodeling and hypertrophy, as well as cardiac arrhythmias, thereby resulting in an increased long-term risk of new-onset heart failure

Experimental and clinical studies also support the production of specific mediators from the steatotic/inflamed/fibrotic liver in patients with NAFLD (Fig. 2) [52, 53]. When NAFLD occurs, liver fat and inflammation progress (NASH) and advanced fibrosis develops. In this context, many alterations take place into the liver, resulting in increased production of atherogenic lipids, exacerbation of systemic and hepatic insulin resistance, activation of the renin-angiotensin-aldosterone system and release of several proinflammatory cytokines, pro-oxidant factors and thrombogenic molecules (e.g., IL-6, factor VII, plasminogen activator inhibitor-1, endotelin-1) [8, 9•, 52, 53]. According to the lipotoxicity theory [54], it is likely that there is a pathogenic “cross-talk” between NAFLD and the expanded and inflamed visceral adipose tissue. From this perspective, epicardial adipose tissue (EAT) thickness may represent a marker of the cumulative effects of NAFLD and insulin resistance in the setting of ectopic fat accumulation. It has been shown that the severity of NAFLD is significantly associated with increased EAT thickness, which is in turn associated with LV diastolic dysfunction [55, 56]. In patients with NAFLD, all these factors may also have an adverse effect on the long-term risk of cardiac complications, including the risk of new-onset HF, especially HFpEF [8, 9•].

Finally, some genetic polymorphisms predisposing individuals to advanced forms of NAFLD, such as *patatin-like phospholipase domain-containing protein-3* (PNPLA3) and *trans-membrane 6 super family-2* (TM6SF2), may modulate the association between NAFLD and the risk of cardiovascular disease [8, 9•, 57]. For instance, the *PNPLA3* rs738409 C > G and *TM6SF2* rs58542926 C > T are two genotypes that increase the risk of developing advanced forms of NAFLD [57], but may also promote the reduction in plasma very-low-density lipoprotein (VLDL) concentrations, thereby reducing the risk of cardiovascular disease in patients with NAFLD [57]. However, at present, it is uncertain if the *PNPLA3* rs738409 C > G or *TM6SF2* rs58542926 C > T genetic variants might also modulate the association between NAFLD and the risk of new-onset HF [9•].

## Pharmacological Treatments that Beneficially Affect NAFLD and HF

Lifestyle modifications, which include hypocaloric diet and physical activity to achieve weight loss, are the cornerstone of treatment for NAFLD [5, 12]. A weight loss of ≥ 10% is associated with resolution of NASH, and significant improvement of liver fibrosis. Moderate weight loss (ranging from ~ 5% to 10%) may also improve various histologic components of the NAFLD activity score [58]. For such reason, the European and American practice guidelines for the management of NAFLD recommend that in overweight or obese patients with NAFLD, a 5–10% weight loss is the goal of most lifestyle interventions [12, 18•]. Among the different therapeutic options to lose body weight, bariatric surgery is currently the most effective strategy [59]. In severely obese patients with NAFLD, bariatric surgery can improve all histological features of NASH, including also liver fibrosis [59]. In a systematic review and meta-analysis of 32 cohort studies including 3,093 liver biopsy specimens, Lee et al*.* reported that bariatric surgery procedures resulted in histologic resolution of hepatic steatosis in ~ 65% of patients, hepatocyte ballooning in ~ 75% of cases and liver fibrosis in ~ 40% of cases [60]. In a recent multicentre, open-label, randomised clinical trial enrolling 288 severely obese patients with biopsy-proven NASH, who were randomly assigned to lifestyle modification *plus* medical care, Roux-en-Y gastric bypass, or sleeve gastrectomy, Verrastro et al*.* reported that the percentage of patients who met the histological resolution of NASH without fibrosis worsening, at 1-year follow-up, was significantly greater in the Roux-en-Y gastric bypass group (56%) and the sleeve gastrectomy group (57%) when compared with the nonsurgical group (16%) [61]. Among patients with NASH and obesity, bariatric surgery, compared with nonsurgical management, was also associated with a significantly lower risk of major adverse cardiovascular and liver-related outcomes [62, 63]. Emerging evidence indicates that bariatric surgery might be also considered for treating severely obese patients with advanced HF [9•], as this surgical procedure is able to improve cardiac structure and function [64–66].

Currently, there are no approved pharmacotherapies for NAFLD and its more advanced forms. The current scientific guidelines for the NAFLD management recommend the use of pioglitazone in patients with biopsy-proven NASH and/or advanced fibrosis, regardless of the presence or absence of T2DM [5, 12, 18•]. However, safety concerns due to moderate weight gain, fluid retention and peripheral oedema limit the use of pioglitazone in clinical practice that should be avoided in patients at high risk of HF. Indeed, current HF guidelines do not recommend the use of pioglitazone in patients with symptomatic HF or in those at high risk of HF [1].

**Fig. 3:**
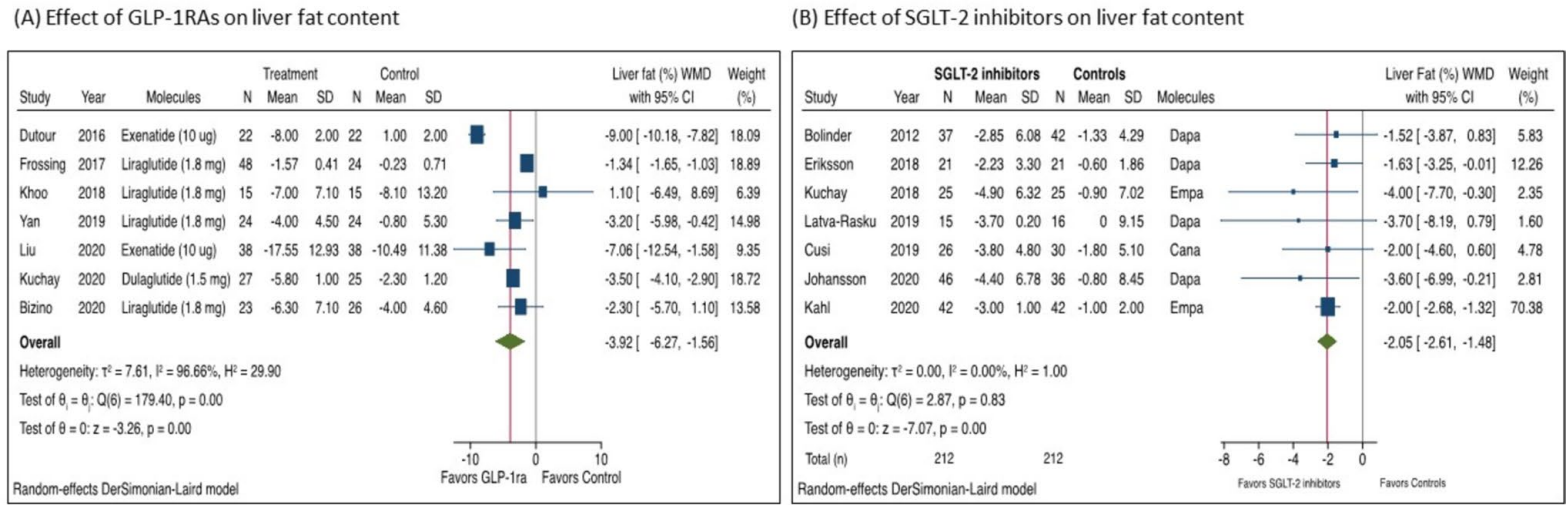
(**A**) Forest plot and pooled estimates of the effect of GLP-1RAs on the absolute percentage of liver fat content as assessed by magnetic resonance-based techniques (*n* = 7 randomized controlled trials) when compared with placebo or reference therapy. Data are reproduced from Mantovani et al. [68]. (**B**) Forest plot and pooled estimates of the effect of SGLT-2 inhibitors on the absolute percentage of liver fat content as assessed by magnetic resonance-based techniques (*n* = 7 randomized controlled trials) when compared with placebo or reference therapy. Data are reproduced from Mantovani et al. [69].

Growing clinical evidence indicate that glucagon-like peptide-1 receptor agonists (GLP-1RAs) and sodium-glucose cotransporter-2 (SGLT-2) inhibitors have hepatoprotective effects [67–69, 70•], as well as beneficial effects on the long-term risk of adverse cardiovascular and kidney outcomes, regardless of T2DM status [71, 72]. GLP-1RAs are approved for the treatment of T2DM. These glucose-lowering agents improve insulin resistance and promote body weight loss. For such reason, GLP-1RAs have been extensively studied in patients with NAFLD or NASH. A recent meta-analysis of 11 placebo-controlled or active-controlled phase-2 randomized controlled trials (RCTs) (involving a total of 936 middle-aged individuals) showed that compared with placebo or reference therapy, treatment with GLP-1RAs for a median of 26 weeks was associated with a significant reduction in the absolute percentage of liver fat content assessed by magnetic resonance-based techniques (pooled weighted mean difference: -3.92%, 95% CI -6.27% to -1.56%) (Fig. 3, panel A) and serum liver enzyme levels, as well as with greater histological resolution of NASH without worsening of fibrosis (pooled random-effects odds ratio 4.06, 95% CI 2.52–6.55; for subcutaneous liraglutide and semaglutide only) [68]. In this meta-analysis, GLP-1RA treatment was not associated with an improvement in liver fibrosis on histology [68]. GLP-1RAs have also beneficial effects on all-cause mortality and cardiovascular and kidney outcomes. For instance, a 2019 meta-analysis of seven RCTs for a total of nearly 56,000 individuals with T2DM showed a significant reduction of the risk of major adverse cardiovascular events (defined as cardiovascular mortality, nonfatal stroke or myocardial infarction), all-cause mortality, hospital admission for HF and worsening of kidney function [71]. Specifically, GLP-1RAs significantly reduced hospital admission for HF by nearly 10% (HR 0.91; 95% CI 0.83–0.99) [71]. However, it should be noted that a recent network meta-analysis aimed at evaluating GLP-1RAs and SGLT-2 inhibitors in patients with T2DM at varying cardiovascular risk reported that GLP-1RAs had a little or even no effect on hospital admission for HF (odds ratio 0.94, 95% confidence interval 0.85–1.03) compared with SGLT-2 inhibitors [72]. GLP-1RAs are usually well tolerated in clinical practice, although these drugs may induce nausea, constipation, abdominal pain or diarrhea, especially in the first weeks of use. Thad said, GLP-1RAs appear to be a valuable option for the treatment of NAFLD patients with or without coexisting HF (Table 1). However, given that there are no data from large RCTs with liver histological endpoints, the practice guidelines released from the European and American hepatology societies for management of NAFLD did not yet recommend the use of GLP-1RAs to specifically treat NAFLD or NASH [18•, 73•].

**Table 1:** Pharmacological treatments that may beneficially affect NAFLD and HF

Drug Class	NAFLD benefits	HF benefits	Comments
GLP-1RAs *(e.g., exenatide, liraglutide, dulaglutide, semaglutide)*	Evidence of improving histological features of NASH (steatosis, ballooning, lobular inflammation) or achieving resolution of NASH without worsening of liver fibrosis	Small reduction of hospitalization for HF in patients with T2DM	Promising therapeutic option to specifically treat NAFLD or NASH, but uncertain use for patients with established HF
SGLT-2 inhibitors *(e.g., empagliflozin, dapagliflozin, canagliflozin)*	Evidence of improving hepatic fat content on imaging techniques	Reduction of hospitalization for HF and CVD death, regardless of the presence of T2DM and left ventricular ejection fraction	Promising therapeutic option to specifically treat NAFLD or NASH in patients with concomitant HF
ACE-inhibitors or ARBs or ARNI	No clear evidence of improving histological features of NAFLD	Reduction of hospitalization for HF and CVD death, especially in patients with HFrEF	Large RCTs are required to provide data on possible hepatoprotective effects in patients with NAFLD or NASH

*ACE* angiotensin-converting enzyme, *ARB* angiotensin II receptor blocker, *ARNI* angiotensin receptor-neprilysin inhibitor, *CVD* cardiovascular disease, *GLP-1RAs* glucagon-like peptide 1 receptor agonists, *HF* heart failure, *HFrEF* heart failure with reduced ejection fraction, *NAFLD* non-alcoholic fatty liver disease, *NASH* non-alcoholic steatohepatitis, *RCT* randomized controlled trial, *SGLT-2* sodium-glucose cotransporter-2, *T2DM* type 2 diabetes mellitus

SGLT2 inhibitors are a relatively newer class of glucose-lowering drugs that act mainly by inhibiting SGLT2 receptors in the proximal convoluted tubule of the glomeruli, thus preventing sodium and glucose reabsorption and promoting their excretion in urine [74]. SGLT2 inhibitors are responsible for critical paradigm shifts in the management of patients with or at high risk for HF [75]. In this regard, a recent meta-analysis of 8 RCTs showed that SGLT-2 inhibitors significantly reduced all-cause mortality, cardiovascular mortality and hospitalization for HF [76]. Specifically, in that meta-analysis, treatment with SGLT-2 inhibitors reduced the risk of hospitalization for HF by nearly 30% (HR 0.69; 95% CI 0.64–0.74) [76]. A post-hoc analysis of the DECLARE-TIMI 58 trial also reported that dapagliflozin reduced the risk of first and total non-elective hospitalizations for any cause in patients with T2DM (irrespective of the presence of atherosclerotic cardiovascular disease), including hospitalizations not directly attributed to cardiac, kidney, or metabolic causes [77]. Other observational studies and some meta-analyses have even reported that SGLT2 inhibitors are able to reduce the risk of hospitalizations for HF in a broad range of patients with HF, regardless of T2DM status, LVEF and care setting [78–80]. Interestingly, experimental data also reported several favorable effects of SGLT-2 inhibitors on hepatic steatosis, necroinflammation and fibrosis, because of the combination of negative energy balance and substrate switching towards lipids as source of energy. Interestingly, a 2021 meta-analysis of 12 RCTs (involving a total of 850 overweight or obese adults with NAFLD, most of whom had T2DM) examining the efficacy of SGLT-2 inhibitors to specifically treat NAFLD reported that compared to placebo or reference therapy, the treatment with SGLT-2 inhibitors for a median of 24 weeks was associated with a significant improvement in serum liver enzyme levels and in the absolute percentage of liver fat content on magnetic resonance-based techniques (pooled weighted mean difference: -2.05%, 95% CI -2.61 to -1.48%) (as shown in Fig. 3, panel B) [69]. SGLT-2 inhibitors are usually well tolerated in clinical practice, although these agents may induce fungal urinary tract infections, especially in postmenopausal women. However, given that RCTs with histological liver endpoints are not available to date, it is still premature to recommend the use of SGLT-2 inhibitors for the treatment of NAFLD or NASH [18•, 73•]. That said, these findings suggest that SGLT-2 inhibitors might be an attractive therapeutic option in NAFLD patients with or at high risk for HF (Table 1).

Other drugs that are widely used in patients with HF [81], such as angiotensin converting enzyme inhibitors (ACE-inhibitors), angiotensin II receptor blockers (ARBs) and mineralocorticoid receptor antagonists, might exert some hepatoprotective effects in patients with NAFLD. Experimental and clinical studies, although not all, have reported that treatment with ACE inhibitors or ARBs may exert some anti-fibrotic effects on the liver [82–87]. More recently, in a post-hoc analysis of the PARADIGM-HF trial that included 8,232 HF patients with reduced LVEF who had available measures of liver function, treatment with sacubitril/valsartan has shown to significantly improve serum liver enzyme concentrations compared to enalapril after randomization [88]. However, these results should be primarily interpreted as consequence of the beneficial hemodynamic effects of sacubitril/valsartan on increased hepatic congestion, mainly due to elevated central venous pressure occurring in HF patients with reduced LVEF, instead of a drug-induced beneficial effect on hepatic steatosis. Although these classes of anti-hypertensive drugs are not specifically approved for the treatment of NAFLD or NASH, they can be safely prescribed for conventional indications.

## Conclusions

This review further reinforces the notion that NAFLD is a “multisystem disease” mainly affecting the heart and vascular system and interacting with the regulation of several metabolic pathways [41]. Convincing evidence indicates that NAFLD is a risk factor for atherosclerotic cardiovascular disease, which is the leading cause of mortality in people with NAFLD [6, 7]. In the last years, there is a growing body of evidence also supporting a significant association between NAFLD and higher risk of developing new-onset HF.

Although there are multiple potential pathophysiological mechanisms by which NAFLD may adversely affect cardiac function and structure and increase the long-term risk of new-onset HF, no studies to date have proven a cause-and-effect relationship, and further research is certainly needed to better decipher the existing but complex link between NAFLD and risk of new-onset HF.

In the meantime, we believe that the major clinical implications of these findings are that a diagnosis of NAFLD can identify a subset of individuals, who are most exposed to a greater risk of developing both cardiovascular events and new-onset HF. Therefore, individuals with NAFLD might benefit from more intensive surveillance and early pharmacological interventions to decrease the risk of developing these adverse cardiovascular outcomes. As regards to this, future high-quality intervention studies are required to evaluate whether improvement or resolution of NAFLD achieved by treatment with GLP-1RAs and SGLT-2 inhibitors (alone or in combination) may also reduce the long-term risk of new-onset HF.
